# Prophylactic chemotherapeutic hyperthermic intraperitoneal perfusion reduces peritoneal metastasis in gastric cancer: a retrospective clinical study

**DOI:** 10.1186/s12885-020-07339-6

**Published:** 2020-08-31

**Authors:** Lucheng Zhu, Zhizheng Xu, Yajun Wu, Pengyuan Liu, Jianing Qian, Shuhuan Yu, Bing Xia, Jianjun Lai, Shenglin Ma, Zhibing Wu

**Affiliations:** 1 Department of Radiotherapy, Key Laboratory of Clinical Cancer Pharmacology and Toxicology Research of Zhejiang Province, Hangzhou Cancer Hospital, Hangzhou, 310002 P.R. China; 2 Department of Oncology, Key Laboratory of Clinical Cancer Pharmacology and Toxicology Research of Zhejiang Province, Affiliated Hangzhou First People’s Hospital, Zhejiang University School of Medicine, Hangzhou, 310006 P.R. China; 3 Department of oncology, Changxing people’s hospital, Huzhou, 313100 P.R. China; 4 The Second Clinical Medical College of Zhejiang Chinese Medical University, Hangzhou, 310053 P.R. China; 5 Department of Oncology, Zhejiang Hospital, Hangzhou Zhejiang, 310013 P.R. China; 6 Department of Oncology, Jiande Second People’s Hospital, Zhejiang, 311604 P.R. China

**Keywords:** Chemotherapeutic hyperthermic intraperitoneal perfusion, Hyperthermia, Peritoneal metastasis, Microsatellite instability, Gastric cancer

## Abstract

**Background:**

Peritoneal metastasis is the most frequent failure in gastric cancer. This study evaluated the role of prophylactic chemotherapeutic hyperthermic intraperitoneal perfusion (CHIP) in patients after D2 dissection.

**Methods:**

Gastric cancer patients after D2 dissection were enrolled in this study. Patients received either chemotherapy (IV group) or CHIP (CHIP group). Sites of recurrence or metastasis, disease-free survival (DFS), overall survival (OS) and adverse events were evaluated.

**Results:**

Twenty-two patients received CHIP treatment, and 21 patients received chemotherapy alone. The median DFS time was 24.5 and 36.5 months in the IV group and CHIP group (*P* = 0.044), respectively. The median OS time was 33.1 months in the IV group and not reached in the CHIP group (*P* = 0.037). We also found that CHIP could reduce the total recurrence/metastasis rate, especially that of peritoneal metastasis. In the subgroup analysis, DFS and OS were both superior in deficient mismatch repair (dMMR) patients than in proficient MMR (pMMR) patients.

**Conclusion:**

This hypothesis-generating study indicates that CHIP might be feasible for gastric cancer patients after D2 resection.

## Background

Gastric cancer is a life-threatening disease, and each year nearly half of new cases occur in China [1]. After radical surgery, 20–50% of patients still experience peritoneal recurrence or failure [2, 3]. Currently, there is no effective treatment for peritoneal metastasis (PM); therefore, exploration of a proper treatment becomes necessary to reduce or prevent peritoneal recurrence in those high-risk population.

Hyperthermic intraperitoneal chemotherapy (HIPEC) is a promising technique, and several studies have demonstrated its benefit in advanced gastric cancer [4, 5]. HIPEC is also treated as a curative modality in digestive and primary PM [6, 7]. The value of prophylactic HIPEC after D2 dissection is still unknown. GASTRICHIP is an ongoing phase 3 trial designed for prophylactic HIPEC (CHIP), but the results have not been reported [8]. We previously reported that CHIP could reduce peritoneal metastasis [9]. HIPEC could efficiently eliminate tumor cells and induce an anticancer immune response via heat shock protein 90 [10]. Moreover, emerging mechanisms on antitumor immune responses by hyperthermia have been studied, such as immune cells response, damage response of tumor cells, changes of tumor surface molecule and tumor vasculature, and exosomes release [11]. The mismatch repair (MMR) is a system function to keep DNA integrity and genomic stability [12]. Several studies have demonstrated that patients with a deficient MMR (dMMR) status will benefit from immunotherapy [13–15], as it is hypothesized that dMMR tumors can produce additional neoantigens to activate immune cells. In the present study, we address whether gastric cancer patients can benefit from CHIP after D2 dissection and explore the immunogenic effect of hyperthermia.

## Methods

### Study population

This was a retrospective study conducted in Hangzhou Cancer Hospital between Jul 1, 2018 and Dec 31, 2019. Eligibility criteria, as reported previously [9], mainly included histologically proven resectable gastric cancer after D2 dissection; stage IIA to IIIC; and patients received ≥2 cycles chemotherapy with or without CHIP after surgery. All data/samples were fully anonymized before data processing and all patients agreed their medical records used in research.

### MMR protein assessment

3-4 μm sections were prepared and stained for the following proteins (mutL homologue 1, MLH1; mutS homologue 2, MSH2; mutS homologue 6, MSH6; PMS1 homologue 2, PMS2). Immunohistochemical staining for hMSH2 (FE11, Calbiochem™, Cambridge, Massachusetts, USA) and hMLH1 (G168–728, Pharmingen, San Diego, California, USA) was performed according to a previous publication [16]. Immunohistochemical staining for hMSH6 (EP49, Agilent Technologies, Santa Clara, CA) and hPMS2 (EP51, Agilent Technologies, Santa Clara, CA) was performed according to a previous publication [17]. Lymphocytes and normal epithelium served as positive internal controls. The definition of dMMR status was identical to previous publication [18].

### Treatment

Each patient received treatment four to 6 weeks after surgery. Twenty-one patients received intravenous 5-fluorouracil (500 mg/m^2^) and LV (200 mg/m^2^) on days 1 to 5, and intravenous cisplatin (25 mg/m^2^) on days 1 to 3 (IV group); the other 22 patients received the same dose of 5-fluorouracil and LV on days 1 to 5, but with intraperitoneal cisplatin (75 mg/m^2^) on day 1 (CHIP group). The regimen was repeated every 3 weeks for no less than two cycles.

### CHIP procedure

The procedure was carried out simultaneously on day 1 in the CHIP group. First, a Tenckhoff catheter was placed at McBurney’s point. A total of 2000 ml of 45 °C-heated physiological saline consisting of cisplatin (75 mg/m^2^) was continuously irrigated into the abdominal space via the catheter for approximately 30 min. The irrigated solution was absorbed by self-absorption. Abdominal regional radiofrequency hyperthermia was performed immediately after intraperitoneal perfusion with cisplatin via NRL-001 (Jilin Maida Co., Jilin, China) radiofrequency heating device. The intraperitoneal temperature was maintained at 42 ± 0.5 °C by real-time monitoring, and heating was continued for 60 min.

### Efficacy and toxicity

Disease-free survival (DFS, defined as interval between surgical resection to relapse) and overall survival (OS, defined as interval between surgical resection to the date of death or the date of the last follow-up) were evaluated. The adverse events were also evaluated (NCICTC 3.0).

### Analysis of immune cells in peripheral blood

To analyze immune function, peripheral venous blood was obtained from patients before and after two cycles of chemotherapy or CHIP. Serum was aliquoted by centrifugation (200×g for 10 min) at room temperature, and a flow cytometric assay was used to analyze the lymphocyte subpopulations.

### Statistical analysis

The chi-square or Student’s t test was chosen for different variables. Kaplan-Meier was used for survival analyses. Subgroup analyses were used to compare DFS and OS according to the MMR status, TNM stage, and treatment group. Data analyses were performed by IBM SPSS version 22.0 (IBM SPSS, Inc., Chicago, IL, USA) and figure presentations were performed by GraphPad Prism, Version 7.01 (GraphPad Software, Inc., San Diego, CA, USA). All tests were two sided and *P* value < 0.05 was considered significant.

## Results

### Patient characteristics

A total of 43 patients were finally included (Supplementary Fig. S1). By June 28, 2018, the median follow-up time was 58 months (range, 6 to 80 months). Of the 43 patients with surgically resected gastric cancer, 22 received CHIP treatment, and 21 patients received chemotherapy alone. The patient characteristics are shown in Table 1. Both groups had more male patients. Adenocarcinoma was the major histology in both groups (CHIP: *n* = 14, 63.3%; IV; *n* = 15, 71.4%).

**Table 1:** Patient characteristics

Item	CHIP group (***n*** = 22)	IV group (***n*** = 21)	***P*** value
Age (years), median	51 (38–69)	55 (43–68)	0.552
Sex			0.586
Male	14	15	
Female	8	6	
Comorbidities			
Hypertension	10	6	0.252
Hepatitis B	2	2	1
Diabetes	2	2	1
Chronic pulmonary disease	3	4	0.689
Poststroke	1	0	
Coronary heart disease	1	1	
Pathological type			0.804
Adenocarcinoma	14	15	
Signet ring cell carcinoma	6	5	
Mucinous adenocarcinoma	2	1	
Stage			0.876
II	4	5	
IIIA	9	8	
IIIB	6	4	
IIIC	3	4	
MMR status			0.729
dMMR	4	3	
pMMR	18	18	
Surgery mode			0.658
Subtotal gastrectomy	13	11	
Total gastrectomy	9	10	

*CHIP* chemotherapeutic hyperthermic intraperitoneal perfusion; *IV* chemotherapy alone; *dMMR* deficient mismatch repair; *pMMR* proficient mismatch repair

### Treatment outcomes

**Fig. 1:**
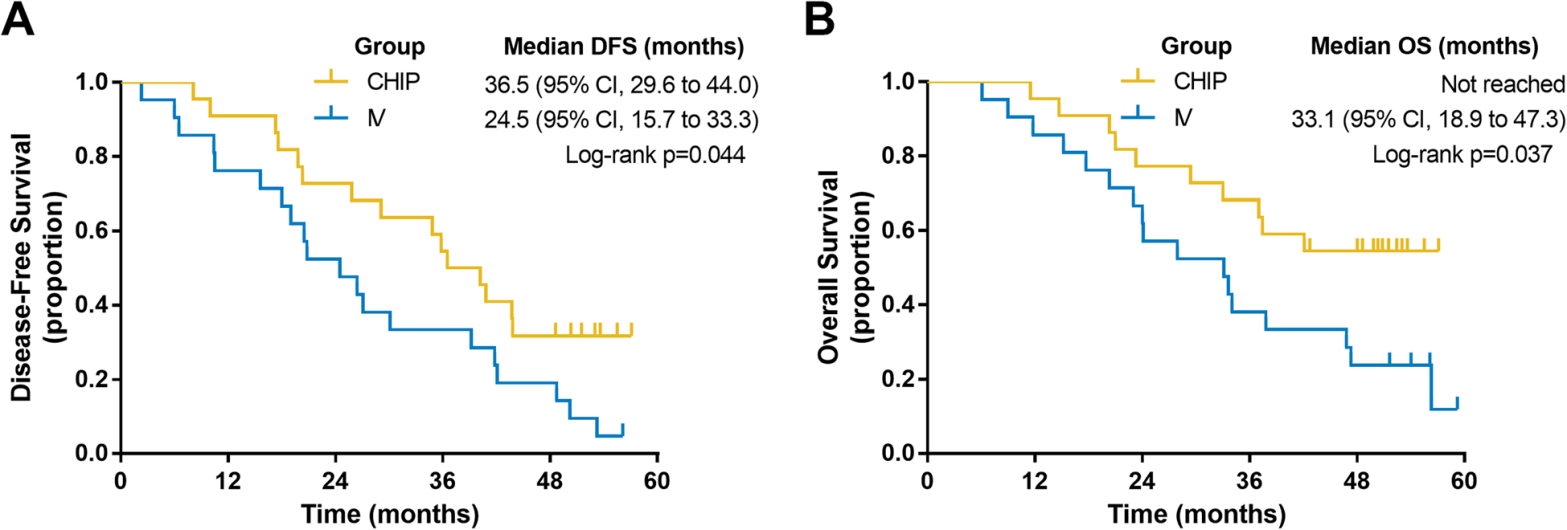
Kaplan-Meier plots of (**a**) disease-free survival (DFS) and (**b**) overall survival (OS) in the CHIP and IV groups. *CHIP* chemotherapeutic hyperthermic intraperitoneal perfusion; *IV* chemotherapy alone

DFS and OS were analyzed between the two regimens (Fig. 1). The median DFS time was 36.5 months in the CHIP group and 24.5 months in the IV group (*P* = 0.044). The median OS time was not reached in the CHIP group and 33.1 months in the IV group (*P* = 0.037). The benefit in terms of DFS and OS in CHIP patients was significantly superior to that in IV patients. In order to reduce confounding effect of MMR status on survival, a bivariate cox regression incorporating MMR status was analyzed, the hazard ratio of OS for CHIP was 0.443 (95%CI: 0.201 to 0.978; *p* = 0.044) and the hazard ratio of DFS for CHIP was 0.518 (95%CI: 0.264 to 1.017; *p* = 0.056).

**Fig. 2:**
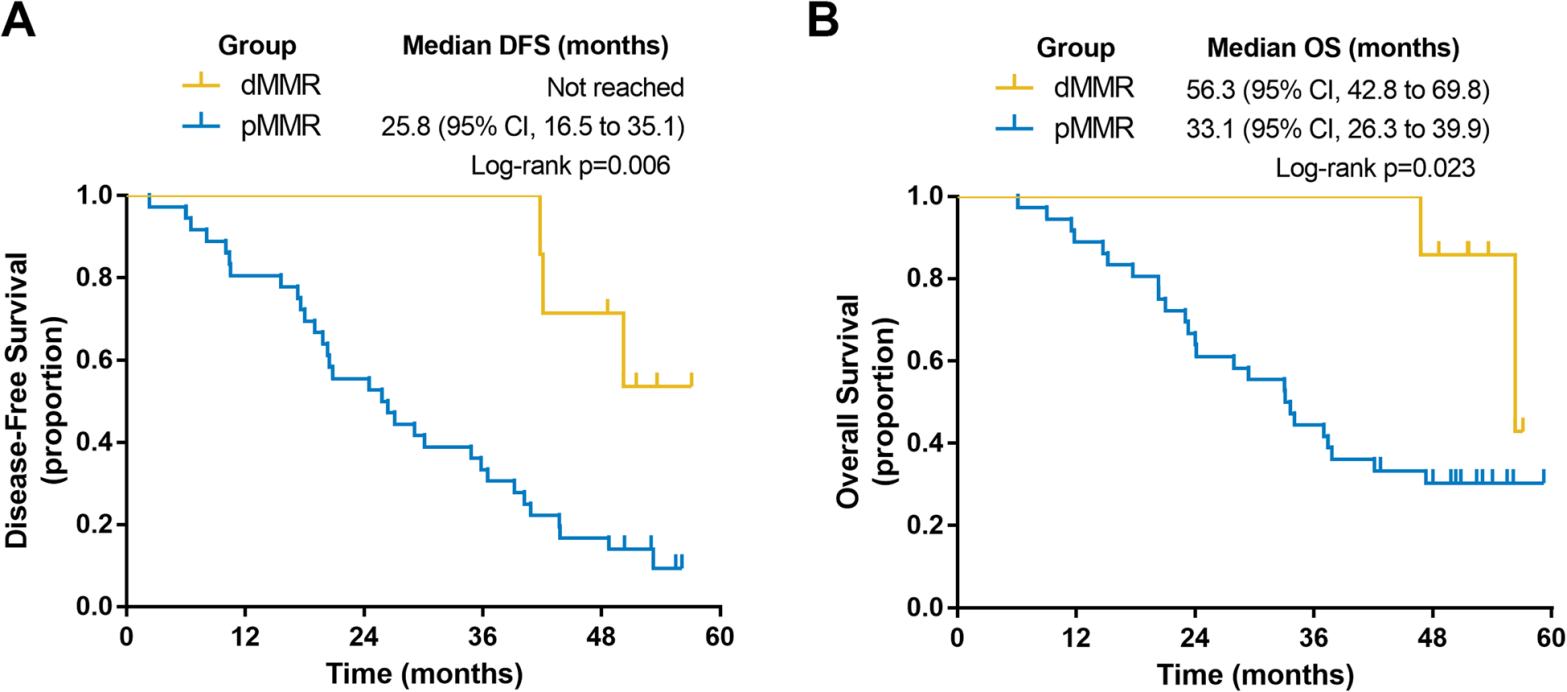
Kaplan-Meier plots of (**a**) disease-free survival (DFS) and (**b**) overall survival (OS) in dMMR and pMMR patients. *dMMR* deficient mismatch repair; *pMMR* proficient mismatch repair

**Fig. 3:**
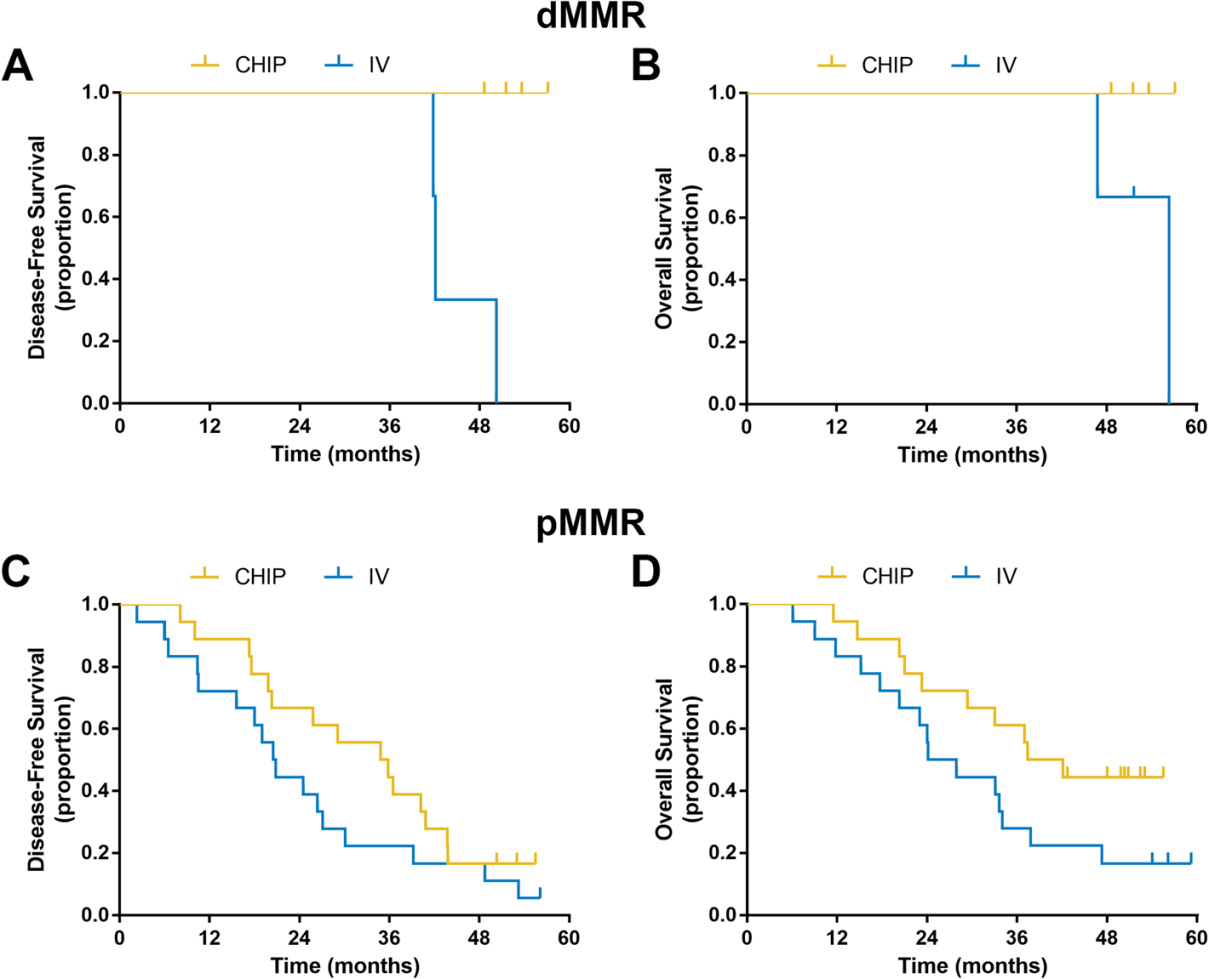
Kaplan-Meier plots of (**a**, **c**) disease-free survival (DFS) and (**b**, **d**) overall survival (OS) in the CHIP and IV groups stratified by the MMR status. *CHIP* chemotherapeutic hyperthermic intraperitoneal perfusion; *IV* chemotherapy alone; *dMMR* deficient mismatch repair; *pMMR* proficient mismatch repair

In the subgroup analysis, both DFS and OS were longer in dMMR patients than in proficient MMR (pMMR) patients (Fig. 2). In dMMR patients, the median DFS time was not reached, and the median OS time was 56.3 months, while in pMMR patients, the median DFS and OS times were 25.8 months and 33.1 months, respectively. To further assess the impact of the MMR status on hyperthermia, we separately analyzed the prognosis in dMMR and pMMR patients (Fig. 3). The median DFS and OS times of dMMR patients in the IV group were 42.1 and 51.6 months, while no dMMR patients who received CHIP show presented recurrence/metastasis or death at the last follow-up. For pMMR patients in the CHIP and IV groups, the median DFS times were 34.8 and 20.5 months, respectively (*P* = 0.184), and the median OS times were 37.4 and 24.1 months (*P* = 0.068), respectively.

### Lymphocyte subsets after treatment

**Fig. 4:**
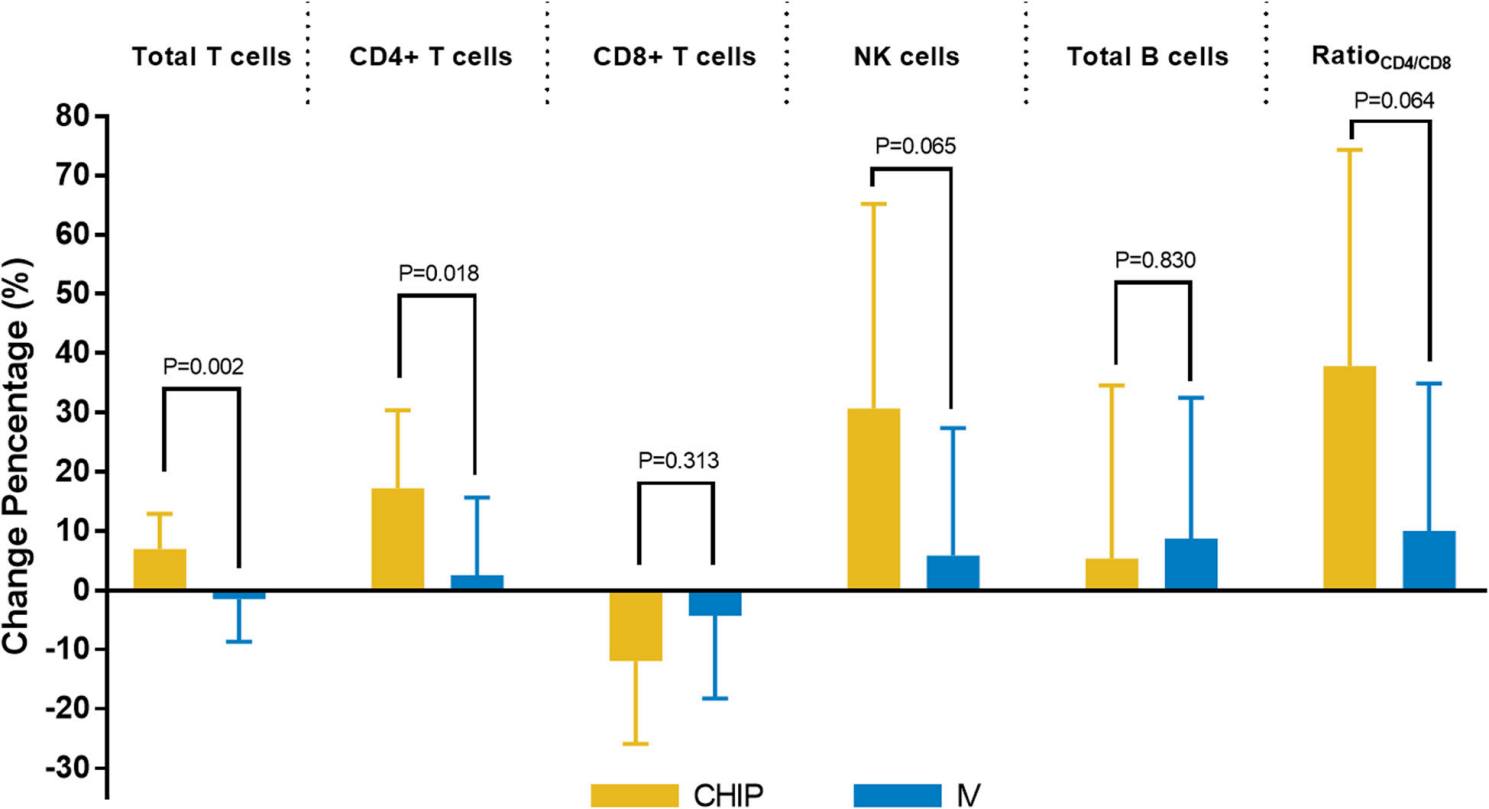
Change in immune cells in peripheral blood after two cycles of treatment in the CHIP and IV groups. *CHIP* chemotherapeutic hyperthermic intraperitoneal perfusion; *IV* chemotherapy alone

As previous studies have reported that hyperthermia could elicit antitumor immune responses, we determined different lymphocyte subsets before and after two cycles of treatment. As shown in Fig. 4, both CHIP and IV treatment could increase/decrease lymphocyte subsets. Compared with IV, CHIP significantly increased the numbers of total T cells and CD4+ T cells. In addition, the number of NK cells was also increased after CHIP, while the statistical value was marginally significant.

**Fig. 5:**
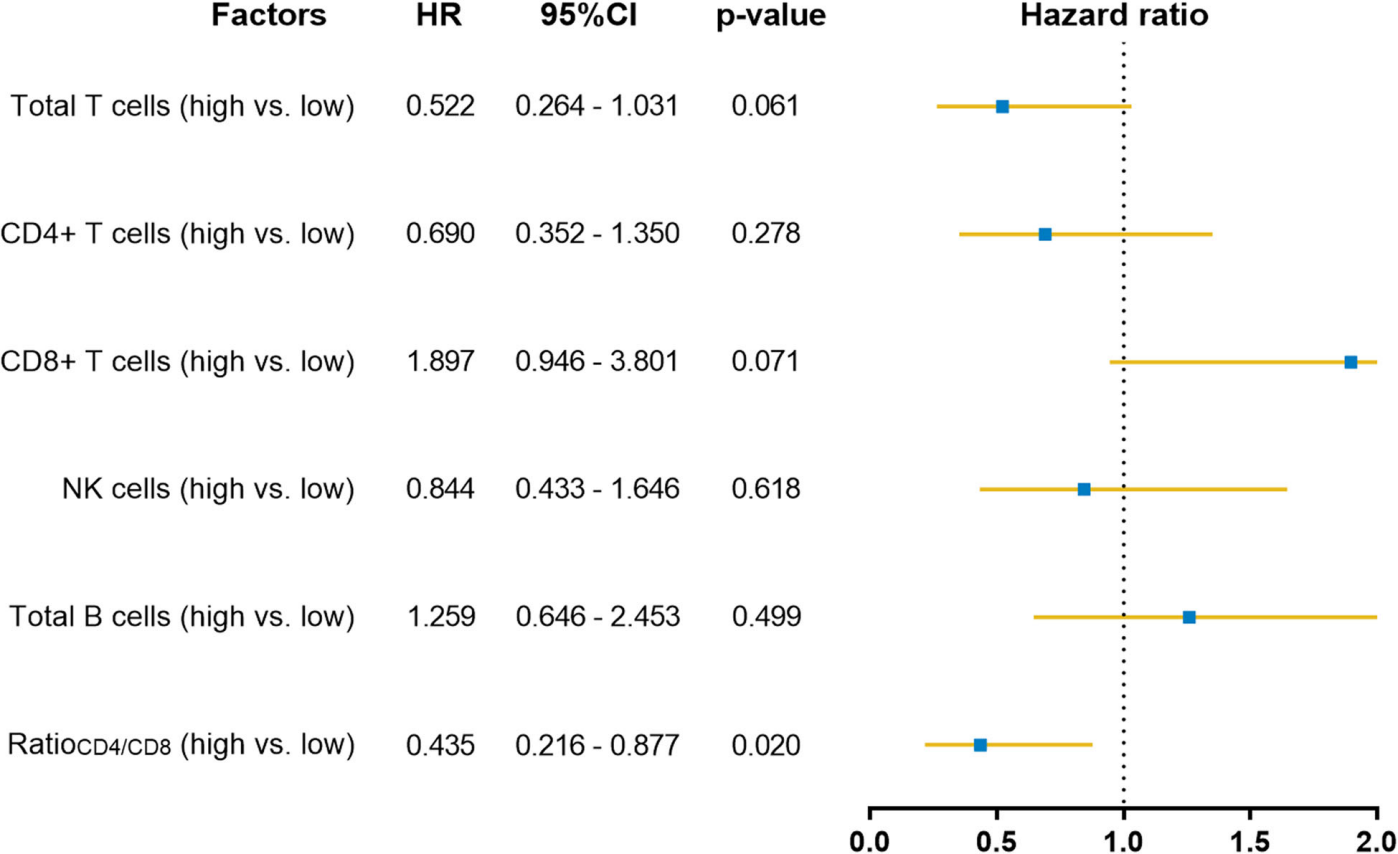
Correlation of changes in immune cells in peripheral blood and disease-free survival (DFS). *The changes in lymphocyte subsets after two cycles of treatment were divided by the median values. High represents values above the median, and low represents values equal to or less than the median*

We observed a survival benefit and shifted lymphocyte subsets after CHIP. Therefore, we evaluated whether the survival benefit correlated with the changes in lymphocytes. The changes in lymphocyte subsets were divided by their median values. As shown in Fig. 5, the ratio of CD4 to CD8 T cells was significantly correlated with DFS (*P* = 0.020). In addition, the numbers of total T cells and CD8+ T cells had marginally significant correlations with DFS.

### Safety and tolerability

The toxicity profiles were similar between the CHIP and IV groups. Details are listed in Table 2. Although myelosuppression occurred more frequently in the CHIP group, it did not reach statistical significance. There was no significantly difference between the two regimens with other grade 3/4 events (*P* > 0.05 for all variables). Interestingly, CHIP had a correlation with a more frequent incidence of grade 1/2 trichomadesis (*P* = 0.021), while IV had a correlation with a more frequent incidence of grade 1/2 chemical peritonitis (*P* = 0.048).

**Table 2:** Toxicities related to treatment in the two groups

Adverse reaction	CHIP group (n = 22)		IV group (n = 21)		***P*** value	
	All grade	Grade 3/4	All grade	Grade 3/4	All grade	Grade 3/4
Leukocytopenia	15	4	9	2	0.095	0.664
Anemia	18	4	12	3	0.078	1.000
Thrombocytopenia	15	7	10	2	0.172	0.132
Trichomadesis	14	2	6	1	0.021	1.000
Liver dysfunction	12	2	7	1	0.161	1.000
Renal dysfunction	8	1	4	0	0.206	1.000
Cardiac dysfunction	4	0	2	0	0.664	–
Nausea/vomiting	18	3	12	2	0.078	1.000
Neurotoxicity	9	0	5	0	0.232	–
Chemical peritonitis	0	0	4	0	0.048	–

*CHIP* chemotherapeutic hyperthermic intraperitoneal perfusion; *IV* chemotherapy alone

### Recurrence pattern

As shown in Table 3, the most common recurrent or metastatic sites were remnant gastric metastasis, retroperitoneal lymph node metastasis, PM, and hepatic metastasis. 63.6% (14 of 22) patients in the CHIP group experienced recurrence or metastases, while 90.5% (19 of 21) patients in the IV group experienced recurrence or metastases (*P* = 0.037). PM (4.5% vs. 33.3%) was less frequent in the CHIP group (*P* = 0.015). Hepatic metastasis (9.1% vs. 23.8%) and seroperitoneum (4.5% vs. 19.0%) were also more frequent in the IV group, but there was no significant difference.

**Table 3:** Recurrence or metastases after treatment

Recurrence or metastasis	CHIP group (n = 22)	IV group (n = 21)	***P*** value
Total	14	19	0.037
Remnant gastric metastasis	5	5	0.933
Retroperitoneal lymph node metastasis	6	7	0.665
Hepatic metastasis	3	6	0.229
Peritoneal metastasis	1	7	0.021
Seroperitoneum	1	4	0.185

*CHIP* chemotherapeutic hyperthermic intraperitoneal perfusion; *IV* chemotherapy alone

## Discussion

In this study, we found that the median DFS and OS times were 36.5 months and more than 50 months, respectively, in the CHIP group, which were significantly longer than those in the IV group. We also found that CHIP could reduce the total recurrence/metastasis rate, especially peritoneal metastasis (4.5% for the CHIP group and 33.3% for the IV group). In addition, in dMMR patients, the median DFS time was not reached and the OS time was 56.3 months, while in pMMR patients, the median DFS and OS times were 25.8 months and 33.1 months, respectively. Further analysis showed that dMMR patients might benefit from CHIP treatment.

The high risk patients developing PM include those with tumors either extending beyond the serosa or that have invaded adjacent structures. Increasing data have shown the feasibility and efficacy of HIPEC in patients with PM [5, 19]; however, the role of CHIP remains inconclusive in patients without PM. A previous study showed that adding CHIP to neoadjuvant chemotherapy could further prolong OS (36 vs. 27 months) compared with neoadjuvant chemotherapy alone [20]. However, another study did not confirm the benefit of CHIP [21]. A recently published study showed that combination of HIPEC and cytoreductive surgery could prolong recurrence-free survival and OS, while it did not result in more side effects compared to surgery alone in ovarian cancer [22]. Our study showed that adding CHIP after D2 resection prolonged DFS and OS. Given these exciting results, CHIP could be used preventing serous cavity carcinomatosis in the future.

The MMR system is the crucial links in suppressing tumor formation and can repair mismatched DNA to keep genome stability [23]. Microsatellite instability (MSI) is reflective of a dMMR status and it can be measured by polymerase chain reaction (PCR) or immunohistochemistry (IHC). This study used IHC to identify MMR status, which could indirectly reflect MSI. MSI indicates the presence of short tandem repeats (commonly 1–6 base pairs) that spread throughout the genome and represents heteromorphosis associating with cancer development. A dMMR status is a positive prognostic factor for survival in patients with stage II colon cancer and might be a negative predictive factor for fluoropyrimidine contained regimens [24, 25]. However, a recent study showed that the dMMR remained a positive predictive factor in stage III colon cancer patients receiving FOLFOX regimen [26]. The role of a dMMR status in gastric cancer remains complicated. Smyth and colleagues performed an exploratory analysis of the MAGIC trial and found that a dMMR status and high MSI were associated with poor survival in patients receiving adjuvant chemotherapy [18]. Another study indicated that a dMMR status was associated with a favorable prognostic role in metastatic gastric cancer patients receiving first-line chemotherapy [27]. Our data showed that patients with a dMMR status had better DFS and OS than those with a pMMR status. A possible explanation for this finding might be that dMMR tumors have a powerful immune cells infiltrate, which could suppress invisible micrometastases after surgery [28, 29]. Chemotherapy might have a negative effect on the body immunosurveillance, thus counteracting the innate benefit of the hypermutated phenotype [18]. In our study, almost all immune cells, including total T cells, CD4+ T cells, and NK cells, were elevated after chemotherapy (there was a minor decrease in CD8+ T cells).

Hyperthermia has been proven to sensitize tumor cells to radio- and chemotherapy and to modulate the immune system [30]. Previous studies have shown improved DFS and local tumor control without an increase in toxicity for the combined treatment. These findings may be partly due to the immunomodulatory function of hyperthermia that has been fully described by Zhang [31]. Fever-like hyperthermia activates both the innate and adaptive immune responses [32]. We also observed an elevation in T cells, CD4+ T cells, and NK cells in both the innate and adaptive immune systems after CHIP treatment. Cisplatin is widely used for the treatment of gastric cancer. It causes cell death through inhibiting and blocking DNA replication and transcription by forming various DNA adducts [33]. Besides inducing DNA damage, cisplatin can also efficiently activated the DNA damage response system [34]. Hyperthermia could enhance intracellular cisplatin accumulation and hamper DNA damage response. The combination of hyperthermia and intraperitoneal cisplatin can lead to an overall enhancement of drug cytotoxicity [35]. Another interesting finding was the different prognoses between dMMR and pMMR patients receiving CHIP. dMMR patients who received CHIP seemed to have better OS and DFS. Compared with pMMR tumors, dMMR tumors usually have an exceptionally higher mutational burden and neoantigen load, as well as more immune cells infiltration [36, 37]. Hyperthermia could activate both the innate and adaptive immune responses. Zunino’s study showed that tumor-specific T cells were activated after tumor cells treated by HIPEC [10]. These data support the hypothesis that high mutant neoantigens in dMMR cancers make them sensitive to CHIP.

This study showed the benefit of adjuvant CHIP. While there were several limitations. This was a retrospective study with small sample size, especially those with a dMMR status. Some patients did not reach observed endpoint, which may have resulted in the efficacy of CHIP not being fully demonstrated.

## Conclusions

We found that CHIP might offer a survival benefit and improved local control in patients with gastric cancer after D2 resection. A dMMR status seems to be a good predictive biomarker for prognosis, especially in patients with immunologically activated disease receiving CHIP. Because data from the present study are preliminary, further studies are is needed to confirm the clinical potential of CHIP in this population.

## Supplementary information

**Additional file 1.**

## Data Availability

The datasets used and/or analyzed during the current study are available from the corresponding author on reasonable request.

## Abbreviations

PM: Peritoneal carcinomatosis
HIPEC: Hyperthermic intraperitoneal chemotherapy
CHIP: Prophylactic hyperthermic intraperitoneal chemotherapy
MMR: Mismatch repair
dMMR: deficient mismatch repair
pMMR: proficient mismatch repair
DFS: Disease-free survival
OS: Overall survival
