# Supplementary figures and images for: Prophylactic chemotherapeutic hyperthermic intraperitoneal perfusion reduces peritoneal metastasis in gastric cancer: a retrospective clinical study
Source: BMC Cancer. 2020 Aug 31;20:827. doi: 10.1186/s12885-020-07339-6 (PMC7461269; doi:10.1186/s12885-020-07339-6)

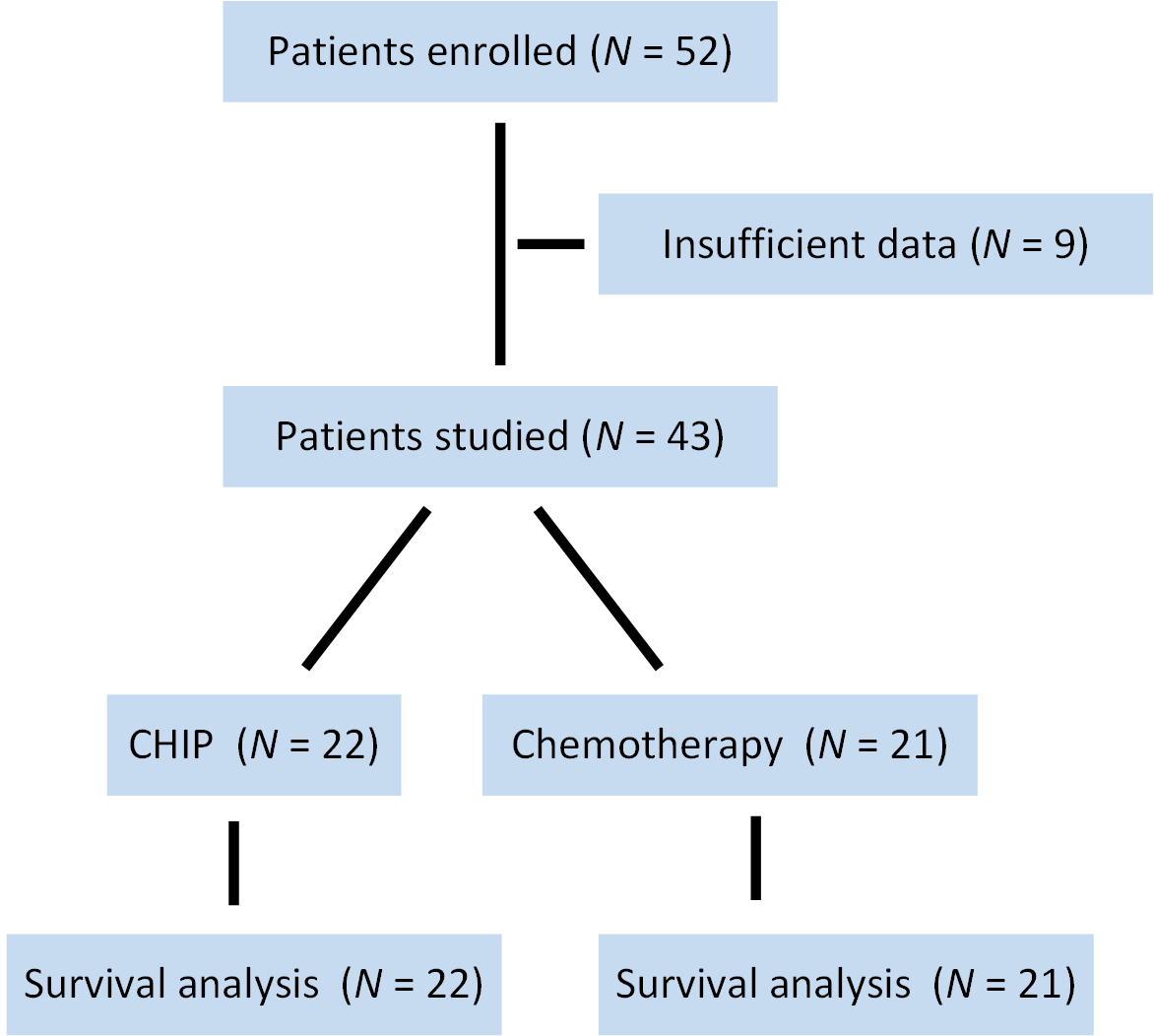

Supplement: Supplementary file 1 — Additional file 1. [file 12885_2020_7339_MOESM1_ESM.tif]
